# Impact of alkaline and deep eutectic solvent extraction on rapeseed protein isolates characteristics and *in vitro* digestibility

**DOI:** 10.1016/j.crfs.2025.101075

**Published:** 2025-05-13

**Authors:** Branislava Đermanović, Aleksandar Marić, Marijana Sakač, Damjana Tomić, Danka Dragojlović, Ljiljana Popović, Bojana Šarić, Pavle Jovanov

**Affiliations:** a Institute of Food Technology in Novi Sad, University of Novi Sad, Bulevar cara Lazara 1, 21000, Novi Sad, Republic of Serbia; b Faculty of Technology, University of Novi Sad, Bulevar cara Lazara 1, 21000, Novi Sad, Republic of Serbia

**Keywords:** Rapeseed cake, Rapeseed protein isolates, Alkaline extraction, DESs, Functionality, *In vitro* digestion

## Abstract

This study examined the efficiency, purity, functional properties, and nutritional quality of rapeseed protein isolates extracted using alkaline extraction (ALK12) and two deep eutectic solvents (DES) systems – choline chloride:urea (DES1) and betaine:citric acid (DES2), chosen to cover distinct pH ranges. Alkaline extraction achieved the highest protein extraction efficiency (36.9 %), while DES1 achieved the highest protein purity (95.8 %). Alkaline extraction resulted in higher levels of polyphenols and tannins, while DES-based extraction led to higher phytic acid content. DES-based isolates exhibited a more balanced amino acid profile, particularly for sulphur-containing amino acids, making them more valuable protein sources compared to alkaline-extracted proteins. Techno-functional analysis favoured ALK12 for products requiring high water and oil absorption capacity, and foam stability, while DES1, despite lower absorption and foaming capacity, offered good solubility making it suitable for a wide range of applications. FT-IR spectroscopy revealed that both ALK12 and DES2 induced significant conformational changes in protein structure, likely due to extreme pH conditions. In contrast, DES1 exhibited a cleaner spectrum and more defined protein peaks, indicating high purity and stable structure. Lab-on-a-chip electrophoresis confirmed a more diverse protein profile for ALK12, while DES1 and DES2 exibited more consistent profiles, suggesting that these systems may preserve higher-order protein structures, such as cruciferin oligomers, and facilitate the extraction of structurally intact proteins. *In vitro* digestibility results showed more efficient enzymatic breakdown for ALK12 and DES1. These findings suggest that the DES1 system could be a promising, sustainable, and efficient alternative to traditional extraction methods. However, future research should focus on optimisation strategies that consider various factors, refining the method for specific applications.

## Highlights

• DES extraction vs. alkaline extraction for rapeseed protein isolate production was compared.
• Alkali extraction showed the highest efficiency.
• Alkali isolate had higher TPC, while DES-based isolates contained more phytic acid.
• Protein secondary structure remained intact in alkali and ChCl:urea isolates.
• Betaine:citric acid isolate had the highest *in vitro* digestibility, followed by ChCl:urea and alkali isolates.

## Graphical abstract

**Figure:**
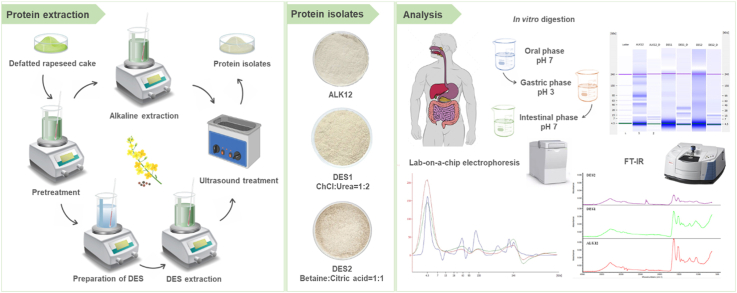

## 1 Introduction

The growing demand for food, driven by population growth, economic development, and changing consumer habits, has placed increasing pressure on global food systems, highlighting the need for sustainable protein sources. As meat production faces resource constraints and environmental challenges (Bryant et al., 2019), the food industry is increasingly shifting towards alternative solutions.

In line with the principles of industrial symbiosis and the circular economy, there is a growing focus on valorising by-products from the edible oil industry as a source of food-grade proteins, particularly those rich in sulphur-containing amino acids with well-balanced essential amino acid (EAA) profile (Joseph et al., 2020; Kumar et al., 2021). These proteins can effectively complement the amino acid profiles of other plant-based sources, making them ideal candidates for the development of high-quality plant-based food products with nutritional value comparable to animal proteins.

In recent decades, both scientific literature and industrial applications have increasingly recognised rapeseed and its by-products as valuable protein sources for food applications (Aider and Barbana, 2011; Chmielewska et al., 2021; Karabulut et al., 2025). The most commonly used method for rapeseed protein extraction involves alkaline treatment (pH 9–12), followed by acid precipitation (pH 2.5–5.5). This process offers several advantages, including high protein yield and the ability to solubilise and recover storage proteins efficiently (Karabulut et al., 2025). However, it also has certain drawbacks, such as the irreversible denaturation of cruciferin and significant loss of napin, reducing the final solubility of rapeseed proteins (Akbari and Wu, 2015). Additionally, during alkaline extraction, the oxidation of phenolic compounds, present in significant amounts in rapeseed meal, generates quinones, reactive intermediates that interact with proteins, leading to dark green or brown discolouration and negatively affecting the nutritional quality and techno-functional properties of rapeseed proteins (Leung et al., 1979; Xu and Diosady, 2002). Given the aforementioned challenges and environmental concerns associated with conventional alkaline extraction, there is a growing interest in developing greener and more sustainable alternatives. In this context, deep eutectic solvents (DES) have gained attention as promising extraction media due to their low toxicity, biodegradability, chemical tunability, cost-effectiveness, and eco-friendly nature (El Achkar et al., 2021; Zainal-Abidin et al., 2017; Zhou et al., 2022). Previous research on the use of DES in rapeseed protein isolation has reported varying results regarding protein yield and purity. Karimi et al. (2024) investigated four formulations of natural deep eutectic solvents (NDESs) and demonstrated that their application resulted in proteins with improved colour and functionality compared to those isolated by alkaline extraction at pH 12, as well as better preservation of the native protein structure than both pH 9 and pH 12 treatments. Among the tested NDES solvents, choline chloride:urea (ChCl:urea) exhibited the highest extraction efficiency, achieving a protein yield of 52.89 % and a protein content of 84.03 %. In contrast, Grudniewska et al. (2018) obtained a significantly lower protein yield of 20 %, with the resulting protein content ranging from 40 % to 50 % by using a different choline chloride-based DES (ChCl/glycerol). These findings underscore the critical role of DES formulation selection in optimising protein recovery and quality.

To further explore the potential of DES in rapeseed protein extraction, this study investigated two distinct DES systems – choline chloride:urea (ChCl:urea) and betaine:citric acid – selected to cover a broader pH range and assess their impact on extraction efficiency and protein quality. By systematically comparing these two solvent systems with the conventional alkaline extraction method, previously optimised by the authors, this study provides insights into the extent to which DES extraction technique is comparable to conventional methods.

In addition to the evaluation of chemical composition, antinutritional factors, colour, molecular and structural characteristics, and techno-functional properties of the resulting protein isolates, the study further examined their amino acid composition and *in vitro* digestibility as crucial parameters for determining nutritional value and potential applications in food formulations. This broader scope offers a more comprehensive assessment of DES suitability for rapeseed protein extraction, addressing key gaps in previous studies that primarily focused on DES-based extraction of rapeseed proteins (Grudniewska et al., 2018; Karimi et al., 2024).

## 2 Materials and methods

### 2.1 Materials

Cold-pressed rapeseed cake was generously provided by Suncokret (Hajdukovo, Serbia). The cake was ground into a fine powder and subsequently defatted twice using n-hexane (Carlo Erba, Vigevano, Italy) at a solid-to-liquid ratio (w/v) of 1:5. The defatting process was conducted at room temperature (23 ± 1 °C) in a beaker with continuous stirring at 1000 rpm for 30 min per cycle. After defatting, the cake was allowed to rest undisturbed for 24 h to ensure the complete evaporation of any residual hexane.

### 2.2 Preparation of DESs

To investigate the influence of different pH values on the protein extraction process, and considering that the acidity and basicity of the extraction medium are important factors affecting both the structural and functional integrity of the extracted proteins, two different DES systems were selected for evaluation. These systems were chosen to represent distinct pH values while avoiding extreme conditions that could potentially contribute to protein denaturation.

The first DES (DES1 system) was composed of choline chloride, ≥98 % (Sigma-Aldrich GmbH, Sternheim, Germany), and urea (Centrohem, Stara Pazova, Serbia) in a molar ratio of 1:2, with 10 % water (w/w). The water content was adjusted to ensure that, at the extraction temperature of 60 °C, DES1 system would achieve a slightly alkaline pH value (7.60). The second DES (DES2 system) was composed of betaine, anhydrous, 98 % (Thermo Fisher, Karlsruhe, Germany), and citric acid (Carl Roth GmbH + Co. KG, Karlsruhe, Germany) in a molar ratio of 1:1. The water content was adjusted to 20 % (w/w) to achieve an acidic pH of 2.39 at 60 °C. Both DES systems were prepared using the heating method described by Dai et al. (2013), with some modifications. The components of each DES were weighed accurately and mixed in a glass beaker. The mixtures were stirred on a magnetic stirrer (C-MAG HS 4, IKA, Germany) at 60 °C for 30 min until clear, homogeneous liquids were obtained. The resulting DESs were then allowed to cool to room temperature prior to further use. The pH of the DESs was measured using a SevenEasy™ pH meter (Mettler Toledo, Urdorf, Switzerland) equipped with an InLab 427 electrode (Mettler Toledo).

### 2.3 Pretreatment for protein extraction

Prior to protein extraction, a pretreatment step involving ultrasound-assisted extraction of phenolics with ethanol was applied to minimise the phenolic content of the defatted rapeseed cake. The defatted rapeseed cake was treated with 84 % ethanol at a solid-to-liquid ratio of 1:60 (w/v). The mixture was stirred on a magnetic stirrer (C-MAG HS 4, IKA, Germany) at 1000 rpm for 30 min. Afterward, the mixture was subjected to ultrasonic treatment in a multi-frequency ultrasonic bath (TI-H-10 2.2gal, Elma Schmidbauer GmbH, Germany) at a frequency of 45 kHz and room temperature for 15 min, with occasional manual stirring using a glass rod. The suspension was then decanted to separate the sediment, which was subsequently used for protein extraction.

### 2.4 Rapeseed protein extraction

#### 2.4.1 DES extraction

Six grams of rapeseed meal were combined with 60 g of solvent in a beaker and placed on a magnetic stirrer (C-MAG HS 4, IKA, Germany). Extraction was performed at 60 °C and 1000 rpm for 2 h. After 1 h, ultrasonic treatment (45 kHz, 60 °C, 15 min) was applied with occasional manual stirring. Stirring then continued for an additional hour, and the suspension was left at room temperature overnight.

The mixture was transferred to a centrifuge bottle and centrifuged at 9000 rpm at 20 °C for 10 min using an Eppendorf 5804R centrifuge (Hamburg, Germany). The supernatant was collected, mixed with ethanol (4 × volume), and stored at −4 °C overnight to promote precipitation. The precipitate was recovered by centrifugation under the same conditions, washed with distilled water, and centrifuged again. This process was repeated until the precipitate was free of non-protein nitrogen, ensuring the complete removal of any traces of the extraction solvents. The final precipitate was frozen and dried using a CHRIST Alpha 1–4 LDplus freeze dryer (Osterode am Harz, Germany) at 0.0128 bar for 24 h before storage. The dried isolate was stored for further analysis.

Extraction with choline chloride:urea (ChCl:urea) resulted in the DES1 isolate, while the use of betaine:citric acid resulted in the DES2 isolate.

#### 2.4.2 Alkaline extraction

Alkaline extraction was performed using the method previously optimised by the authors, which has been proven to achieve high extraction efficiency. For alkaline extraction, the material was suspended in water (1:20 w/v) and the pH was adjusted to 12 using 1 M NaOH (Lach-Ner, Neratovice, Czech Republic), with continuous pH monitoring. After 60 min of extraction, ultrasonic treatment (45 kHz, room temperature, 30 min) was applied with intermittent manual stirring. The suspension was filtered to remove the insoluble fraction, and the precipitates were frozen, dried under the same conditions, and stored for further analysis.

The isolate obtained via alkaline extraction was labeled as ALK12.

### 2.5 Basic chemical composition and protein extraction efficiency

Basic chemical composition of defatted rapeseed meal and rapeseed protein isolates was determined using the standard methods prescribed by the Association of Official Analytical Chemists (AOAC International, 2000), which included moisture (Official Method No. 926.5), protein (Official Method No. 950.36), ash (Official Method No. 930.22), fat (Official Method No. 935.38), and total dietary fibre (Official Method No. 991.43) determinations.

Non-protein nitrogen was measured in the precipitate obtained with 20 % trichloroacetic acid (Sigma-Aldrich GmbH, Sternheim, Germany) using the Kjeldahl (1883) method and calculated using the following equation:

$$\text{Non}-\text{protein}\;N\;\left(\%\right)=\frac{\left(V\times 0.14\times 0.1\times 100\right)}{a\times m}\times 4$$

where:

V – volume of H_2_SO_4_ (mL),

f – factor of H_2_SO_4_,

a – aliquot (25 mL), and

m – weight (g).

Protein extraction efficiency (%) was determined according to the following equation:

$$\text{Protein}\;\text{extraction}\;\text{efficiency}\;\left(\%\right)=\frac{\text{Protein}\;\text{weight}\;\text{of}\;\text{the}\;\text{isolate}}{\text{Protein}\;\text{weight}\;\text{of}\;\text{the}\;\text{meal}}\times 100$$

### 2.6 Antinutritional profile

The total phenolic content (TPC) was measured using the Folin-Ciocalteu method (Singleton et al., 1999) with some modification. For extract preparation, 0.2 g of the sample was mixed with methanol (Carlo Erba, Vigevano, Italy)-water (80:20, v/v) at a solid-to-liquid ratio of 1:20. The mixture was shaken for 30 min using an orbital shaker (PSU-10i, Boeco, Germany) and then sonicated for 20 min in an ultrasonic bath (TI-H Multi Frequency Ultrasonic Bath, Elma Schmidbauer GmbH, Germany). Following centrifugation (Eppendorf 5804R, Hamburg, Germany), 0.2 mL of the extract was diluted with 7.8 mL of distilled water. Subsequently, 0.5 mL of Folin-Ciocalteu's reagent (Sigma-Aldrich GmbH, Sternheim, Germany) and 1.5 mL of sodium carbonate solution (Fisher Scientific Ltd, Loughborough, UK) (20 g/100 mL) were added. The reaction mixture was allowed to stand at 25 °C for 2 h, and its absorbance was measured at 750 nm using a spectrophotometer (Shimadzu UV-1800, Kyoto, Japan). The TPC was quantified using a calibration curve constructed with gallic acid (0–0.25 mg/mL) (Carl Roth GmbH + Co. KG, Karlsruhe, Germany), and results were expressed as gallic acid equivalents (mg GAE/g).

Condensed tannins were quantified using the vanillin method (Herald et al., 2014). Samples were extracted in 1 % HCl (37 %) (Carl Roth GmbH + Co. KG, Karlsruhe, Germany) at 30 °C for 20 min. One milliliter of the extract was mixed with 5 mL of a vanillin (Sigma-Aldrich GmbH, Sternheim, Germany)-HCl solution (1:1), while the control was prepared by combining 1 mL of the extract with 5 mL of 4 % HCl. After a 20-min incubation at 30 °C, the absorbance of the reaction mixtures was measured at 500 nm. A calibration curve was prepared using catechin (0–1 mg/mL), and results were expressed as catechin equivalents (Sigma-Aldrich GmbH, Sternheim, Germany) (mg CAE/g).

The glucosinolate content was determined according to the MSZ-08-1908 (1989) standard. This method involved measuring the absorbance of the Pd-glucosinolate complex at 425 nm. Sinigrin was used as the reference compound to construct a calibration curve.

Phytic acid content was analysed using a Megazyme assay kit (Wicklow, Ireland). One gram of the sample was digested in 20 mL of 0.66 M HCl overnight at room temperature. After digestion, the extract was centrifuged at 13,000 rpm for 10 min, and 0.5 mL of the supernatant was neutralised with 0.5 mL of 0.75 M NaOH. The neutralised sample underwent enzymatic dephosphorylation, and the levels of free and total phosphorus were measured using molybdenum blue reagent at 655 nm. A standard curve based on phosphorus concentrations was used to calculate the phytic acid content by determining the difference between free and total phosphorus levels.

### 2.7 Amino acid profile

The hydrolysis of the samples was performed with 6 M HCl (0.5 % phenol) at 110 °C for 24 h, followed by dissolution in a pH 2.2 loading buffer and filtration through 0.22 μm PTFE filters. The analysis was conducted using the Biochrom 30 Plus Amino Acid Analyser with ion-exchange chromatography (Spackman et al., 1958). Detection was performed at 570 nm, except for proline, which was detected at 440 nm. Amino acids were identified by comparing retention times with standards. Quantification was based on peak areas and standard calibration curves.

### 2.8 Colour analysis

The colour of protein isolates was measured using a Minolta Chromameter (Model CR-400, Minolta Co., Osaka, Japan). The samples were placed in 20-mm-thick holders and measured against a black-and-white background at room temperature.

CIE *L∗a∗b∗* coordinates were recorded, with *L∗* indicating luminance (0–100), *a∗* representing the red/green axis, and *b∗* the yellow/blue axis (−120 to +120). All measurements were performed in triplicate.

### 2.9 Protein molecular and structural characterisation

#### 2.9.1 Fourier-transform infrared spectrum (FT-IR)

The FT-IR spectra were acquired at room temperature using a Nicolet iS10 Fourier transform infrared spectrometer. A small amount of protein powder was compressed into a thin slice approximately 1–2 mm thick for analysis. Measurements were performed within the spectral range of 4000–500 cm^−1^, with a resolution of 4.0 cm^−1^. Data collection and instrument control were carried out using the Omnic 8.1 software (Thermo Fisher Scientific, Waltham, MA).

#### 2.9.2 Capillary electrophoresis

Capillary electrophoresis was performed using an Agilent 2100 Bioanalyser in combination with the Protein 230 (P230) kit. For sample preparation, 10 mg of isolate was dissolved in 1 mL of phosphate-buffered saline (PBS, pH 7.4). Quantification was performed using bovine serum albumin (BSA) dissolved in the same PBS as the standard. The chips were prepared according to the manufacturer's protocol outlined in the Agilent Protein 230 Kit Guide (Agilent Technologies Manual, reference number G2938-90052).

### 2.10 Protein functional characterisation

#### 2.10.1 Protein solubility

Protein solubility was determined following the method described by Popović et al. (2013), with solubility assessed across a pH range of 2–10. To prepare the sample, 10 mg of protein isolate was weighed into an Eppendorf tube, and 1 mL of buffer solution was added. The mixture was then agitated using a Thermo Shaker TS-100C (BioSan, Latvia) at 25 °C for 1 h, with a shaking speed of 900 rpm.

After mixing, the solution was centrifuged at 14,500 rpm for 10 min (Eppendorf MiniSpin Plus, Eppendorf AG, Hamburg, Germany). The supernatant was carefully decanted, and the soluble protein content in the supernatant was quantified using the method of Bradford (1976).

#### 2.10.2 Capacity of water and oil absorption

Water absorption capacity (WAC) and oil absorption capacity (OAC) were determined following the method described by Rodsamran and Sothornvit (2018). For OAC, 100 mg of protein isolate was weighed into an Eppendorf tube, followed by the addition of 0.9 mL of vegetable oil (sunflower oil). The mixture was vortexed for 1 min and then incubated in a Thermo Shaker TS-100C (BioSan, Latvia) at room temperature for 30 min. After incubation, the sample was centrifuged at 4000 rpm for 20 min (Eppendorf MiniSpin Plus, Eppendorf AG, Hamburg, Germany) at room temperature. The oil was removed by inverting the tube, and the remaining sample was weighed.

The same procedure was used to determine WAC, with the exception that 1 mL of water was used instead of oil per 100 mg of isolate. Both WAC and OAC were expressed as grams of water or oil absorbed per gram of isolate.

#### 2.10.3 Foaming capacity and stability

The foaming capacity (FC) and foaming stability (FS) were determined according to the method described by Popović et al. (2013). Solutions for FC and FS determination were prepared at a concentration of 10 mg/mL by mixing 0.5 g of protein isolate with 50 mL of 0.1 M glycine buffer (pH 10) for 30 min. The mixtures were homogenised using an Ultra-Turrax T25 homogeniser (IKA, Germany) at 7000 rpm for 2 min.

The homogenised solutions were transferred into 50 mL graduated cylinders, and the solution volumes were recorded at 0 min (V_0_) and at subsequent time intervals of 1, 10, 30, 60, and 90 min (V_1_). FC was calculated as the ratio of foam volume to total volume and expressed as a percentage. FS was expressed as the FC measured at different time intervals (1, 10, 30, 60, and 90 min). nije usaglašeno sa slikom. Tamo je do 120 min.

#### 2.10.4 Gelling ability

The gelling ability was assessed and expressed as the least gelation concentration (LGC), defined as the minimum concentration required for gel formation. Specific amounts of protein were suspended in 1 mL of phosphate buffer at pH values of 3, 5, and 7, with a buffer concentration of 0.01 mol/L. For all isolates, the final concentrations were prepared at 2 %, 6 %, 8 %, 10 %, 12 %, 14 %, 16 %, and 18 % (w/v). The cuvettes containing the suspensions were heated for 1 h in a boiling water bath and then rapidly cooled to 4 °C. The samples were maintained at this temperature for an additional 2 h.

### 2.11 *In vitro* digestibility

All isolates were digested *in vitro* using the INFOGEST protocol (Minekus et al., 2014) with minor modifications. The digestion process consisted of three consecutive phases, during which the mixture was gently stirred with a magnetic stirrer in a water bath maintained at 37 °C throughout (Velp Scientifica, Usmate Velate (MB), Italy).

Initially, 0.5 g of the protein isolate was mixed with 5 mL of distilled water and 4 mL of simulated salivary fluid (SSF) electrolyte stock solution. To this mixture, 0.5 mL of α-amylase (Sigma-Aldrich GmbH, Sternheim, Germany) was added, followed by 25 μL of 0.3 M CaCl_2_ and 975 μL of distilled water. The pH was adjusted to 7.0 using 1 M HCl and 1 M NaOH as needed. The mixture was then incubated at 37 °C for 2 min to simulate the oral digestion phase.

To the gastric chyme, 8 mL of simulated gastric fluid (SGF) electrolyte stock solution was added, along with 0.04 g of pepsin (AppliChem GmbH, Darmstadt, Germany), 5 μL of 0.3 M CaCl_2_, 1 M HCl to adjust the pH to 3.0, and 1.995 mL of distilled water. The mixture was incubated at 37 °C for 120 min under conditions simulating gastric digestion.

Subsequently, to the gastric chyme, 11 mL of simulated intestinal fluid (SIF) electrolyte stock solution was added, followed by 0.04 g of pancreatin from porcine pancreas (Sigma-Aldrich GmbH, Sternheim, Germany), 40 μL of 0.3 M CaCl_2_, 1 M NaOH to adjust the pH to 7.0, and 8.960 mL of distilled water. This mixture was incubated at 37 °C for 120 min to mimic intestinal digestion. Preliminary studies have shown that this incubation period is sufficient for the complete hydrolytic action of the enzymes. Therefore, the supernatant was separated by centrifugation at 10,000 rpm for 10 min and subsequently frozen until further analysis.

To evaluate the effects of *in vitro* digestion, the percentage (%) of each individual protein fraction within the total identified peaks was calculated using the peak areas on the electropherogram obtained by capillary electrophoresis.

### 2.12 Statistical analysis

The data were processed statistically using the software package XLSTAT 2024 (Lumivero 196 Denver, CO, USA). Results were expressed as mean ± standard deviation. Analysis of variance (ANOVA) and Tukey's HSD test (α = 0.05) were used for comparison of sample means.

## 3 Results and discussion

### 3.1 Characterisation of defatted rapeseed cake

The basic chemical composition of the defatted rapeseed cake revealed a protein content of 37.4 ± 0.16 %, moisture content of 7.55 ± 0.18 %, ash content of 6.08 ± 0.06 %, total fat content of 1.16 ± 0.04 %, and total fibre content of 38.0 ± 1.22 %. Additionally, the evaluation of antinutritional components indicated a total phenolic content of 2.33 ± 0.07 mg GAE/g, a phytic acid content of 1.72 ± 0.02 g/100 g, tannin content of 2.53 ± 0.31 mg CAE/g, and a glucosinolate content of 28.8 ± 4.70 μmol sinigrin/kg.

### 3.2 Protein isolate extraction and characterisation

The protein extraction efficiency and purity of isolates obtained using alkaline extraction (ALK12) and two deep eutectic solvents (DES1: ChCl:urea with a pH value of 7.60 and DES2: betaine:citric acid with a pH value of 2.45) are summarised in Table 1.

**Table 1:** Protein extraction efficiencies, basic chemical profile, and antinutritional components of rapeseed cake and rapeseed protein isolates.

Material	Protein extraction efficiency^a^ (%)	Basic chemical composition^a^			Antinutritional components^a^			
		Protein content (%)	Ash (%)	Total fat (%)	TPC (mg GAE/g)	Phytic acid (g/100 g)	Tannins (mg CAE/g)	Glucosinolates (μmol sinigrin/kg)
**Defatted rapeseed cake**	–	40.5 ± 0.22	6.58 ± 0.03	1.25 ± 0.02	2.57 ± 0.01	1.86 ± 0.07	2.74 ± 0.20	26.0 ± 2.33

**ALK12**	36.9 ± 0.18^a^	86.0 ± 0.32^b^	2.99 ± 0.02^a^	n.d.	1.78 ± 0.03^a^	1.06 ± 0.01^c^	2.19 ± 0.02^a^	n.d.
**DES1**	23.2 ± 0.15^c^	95.8 ± 0.42^a^	0.07 ± 0.01^c^	n.d.	0.61 ± 0.01^b^	1.42 ± 0.01^b^	1.45 ± 0.03^b^	n.d.
**DES2**	31.2 ± 0.20^b^	70.3 ± 0.31^c^	1.35 ± 0.04^b^	n.d.	0.39 ± 0.01^c^	1.50 ± 0.01^a^	1.37 ± 0.01^c^	n.d.

Different letters in each column indicate significant (*p* > 0.05) difference between the isolates.

n.d. – not detected.

ALK12 – isolate obtained after alkaline extraction at pH 12.

DES1 – isolate obtained by DES extraction (choline chloride:urea = 1:2, 10 % H_2_O, pH 7.60).

DES2 – isolate obtained by DES extraction (betaine:citric acid = 1:1, 20 % H_2_O, pH 2.45).

TPC – total phenolic content.

GAE – gallic acid equivalent.

CAE – catehin equivalent.

a On a dry matter basis.

Among the tested extraction systems, ALK12 achieved the highest protein extraction efficiency (36.9 ± 0.20 %), followed by DES2 (31.3 ± 0.18 %) and DES1 (23.2 ± 0.15 %). Alkaline extraction provided the highest efficiency and a satisfactory purity (86.0 ± 0.32 %), due to its ability to enhance protein solubility by disrupting hydrogen bonds and electrostatic interactions within the protein matrix (Karimi et al., 2024). At pH 12, proteins undergo deprotonation, resulting in an increased net negative charge and enhanced repulsion between protein molecules, which facilitates their dispersion into the extraction medium. Additionally, the low viscosity of the alkaline solution promotes mass transfer, further contributing to a higher protein yield. Our protein extraction efficiency for ALK12 aligns with the findings of Kalaydzhiev et al. (2019), who reported a canola protein recovery of 34.9 % at pH 12. Similarly, Zhang et al. (2020) achieved a slightly higher recovery of approximately 50 % under the same conditions.

Although alkaline extraction is a well-established method known for its high extraction efficiency, certain techno-functional properties and the biological value of the obtained proteins may be affected by this treatment. Although DES-based extraction systems can exhibit lower efficiency due to their higher viscosity, which may hinder mass transfer and reduce protein diffusion and overall extraction efficiency, the aim of this study was to explore DES systems with different pH values as alternative extraction media that could contribute to the improvement of specific functional and nutritional properties of the extracted proteins. Therefore, improving the extraction efficiency alone is not a sufficient indicator of the overall effectiveness of the extraction process. It is crucial to consider the impact on both the quality and bioavailability of the proteins, as these factors are essential in determining the suitability of the extraction method for specific applications, including those in the food industry.

Among the DES systems, the betaine:citric acid system exhibited better extraction efficiency, although the purity of the obtained protein (70.3 ± 0.31 %) was significantly (*p* ≤ 0.05) lower compared to the DES1 isolate. For this DES system, it is assumed that the protonation of carboxyl and amine groups in proteins at the low pH characteristic of the system led to increased electrostatic repulsion between protein molecules, thereby preventing protein aggregation and promoting solubilisation (L'hocine et al., 2006). However, this increased electrostatic repulsion likely compromised protein stability, making the proteins more susceptible to denaturation. Denaturation can result in a loss of solubility and cause proteins to precipitate out of solution, ultimately reducing the overall protein yield. Therefore, while the acidic environment in the betaine:citric acid system may promote solubilisation by preventing aggregation, it also leads to decreased extraction efficiency due to reduced protein stability and the potential for protein precipitation at low pH.

Although the ChCl:urea system demonstrated the lowest protein extraction efficiency, the DES1 isolate exhibited the highest purity (95.8 ± 0.42 % on a dry matter (d.m.) basis). The formation of a hydrogen bond network that stabilises protein molecules in solution likely contributed to minimising losses during the extraction process. Additionally, slightly alkaline conditions help preserve the structural integrity of the proteins, preventing degradation processes that commonly occur under more extreme pH conditions. This combination of factors ensures a higher-quality protein isolate, even at the expense of extraction efficiency.

Previous studies on protein extraction using various DES systems have demonstrated significant variations in extraction efficiency and the purity of the resulting products. Grudniewska et al. (2018) extracted canola protein using ChCl/glycerol (1:2) at 60 °C, 100 °C, and 140 °C, achieving yields of 11.5–19.9 % and protein contents of 36–48 % after back-extraction with water and centrifugation. Similarly, Yue et al. (2021) employed ChCl/butanediol isomer DESs to extract oat proteins, reporting yields of 10.9–42.9 % and protein contents of 38.9–57.4 %. Hewage et al. (2024) also highlighted the effectiveness of DES extraction systems, achieving a 65.4 % yield and 92.3 % protein content for fava bean protein extraction using a ChCl/glycerol/water system. Using the same DES system (ChCl:urea with water in a 1:2:1 ratio) and starting material as in this study, Karimi et al. (2024) achieved a significantly higher protein content of 84.03 ± 0.86 % and an extraction efficiency of 52.89 ± 0.55 %. However, these results were obtained through an additional dialysis process conducted over 5 days, which may explain the higher protein yield compared to the present study. An additional advantage of our study, ensuring no overestimation of protein content and extraction efficiency values, is that the samples were thoroughly rinsed during preparation until all non-protein nitrogen, potentially originating from the DES extraction system, was completely removed.

The obtained protein isolates significantly differ in terms of ash content. Specifically, the DES1 isolate exhibited the lowest ash content (0.07 ± 0.01 %), indicating a higher purity of the protein with minimal inorganic material present. In contrast, the DES2 and ALK12 protein isolates showed considerably higher ash content (1.35 ± 0.04 % and 2.99 ± 0.02 %, respectively), suggesting that these systems may have extracted more inorganic compounds along with the proteins. These differences in ash content reflect the varying interactions of the extraction systems with inorganic materials, highlighting the differences in the purity of the protein isolates obtained from each system. Thus, DES1, with its lower ash content, indicates a purer protein isolate, while the higher ash content in DES2 and ALK12 isolates suggests greater solubilisation of inorganic components along with the proteins.

### 3.3 Antinutrient profile of isolates

Rapeseed meal contains antinutritional factors, such as glucosinolates, phytic acid, and polyphenols (sinapine and condensed tannins), which can negatively impact both the nutritional value and sensory acceptance of produced protein isolates (Wanasundara et al., 2017; Zhang et al., 2020, 2024). Polyphenols and phytates interfere with the absorption of proteins and minerals by forming complexes with these nutrients, thereby reducing their bioavailability (Albe-Slabi et al., 2022; Axentii and Codină, 2024; Wanasundara, 2011). Therefore, it is essential to develop protein isolation methods that not only preserve the functional and nutritional integrity of the proteins but also minimise the presence of these antinutritional factors.

Although pretreatment with ethanol and ultrasound was employed in this study as a strategy to reduce antinutrient levels, residual amounts of these compounds remained in the obtained protein isolates (Table 1).

The total phenolic content (TPC) was highest in the ALK12 isolate (1.78 ± 0.03 mg GAE/g), followed by significantly lower values (*p* ≤ 0.05) in the DES1 (0.61 ± 0.01 mg GAE/g) and DES2 (0.39 ± 0.01 mg GAE/g) isolates (Table 1). The higher TPC in the ALK12 isolate can be attributed to the ability of alkaline conditions to enhance polyphenol extraction. At higher pH, the deprotonation of hydroxyl groups in phenolic compounds increases their solubility and facilitates stronger electrostatic interactions with negatively charged proteins, leading to higher retention in the protein isolate (Pasquet et al., 2024). In contrast, DES-based extractions resulted in significantly (*p* ≤ 0.05) lower TPC values, particularly in DES2, where the acidic medium may have contributed to limited solubility and possible degradation of phenolic compounds. The reduced extraction efficiency of polyphenols under acidic conditions can be explained by their tendency to form hydrogen-bonded aggregates, which limit their solubility. Additionally, protonation of functional groups under low pH conditions may have reduced electrostatic interactions between phenolic compounds and proteins, resulting in lower retention in the DES isolates.

The formation of phytic acid-protein complexes is highly pH-dependent, with reduced formation under alkaline conditions (Amat et al., 2024). As a result, phytic acid levels were higher in the DES-based isolates compared to the ALK12 isolate (Table 1). The DES1 system (pH 7.6) enhanced phytic acid solubility through hydrogen bonding and ionic interactions, facilitating its co-extraction with proteins. In contrast, the DES2 system (pH 2.45) further promoted phytic acid solubilisation, leading to higher retention. On the other hand, the alkaline conditions in the ALK12 isolate production likely caused partial precipitation of phytic acid, as it forms insoluble complexes with divalent cations and proteins at higher pH levels. This is consistent with previous studies that reported reduced phytic acid content in alkaline-treated protein isolates (Amat et al., 2024; Karimi et al., 2024).

pH-dependent solubility was also observed for tannins, which are known to form complexes with proteins and affect their digestibility (Cheryan and Rackis, 1980). In an alkaline environment, their deprotonation enhanced solubility and extraction, leading to higher retention in the ALK12 isolate (Table 1). In contrast, tannins in DES1 (pH 7.6) exhibited moderate solubility, whereas the acidic conditions in DES2 (pH 2.45) likely contributed to the lowest tannin content, possibly due to hydrolytic degradation or reduced solubility in the presence of citric acid.

Glucosinolates were not detected in any of the isolates (Table 1).

The results can be summarised by concluding that different extraction methods had distinct effects on the antinutrient composition of the protein isolates. Alkaline extraction resulted in higher levels of polyphenols and tannins, while DES-based extractions showed lower levels of these compounds but higher phytic acid content. These findings align with those observed by Karimi et al. (2024). Based on these results, DES-based extraction may be a more favourable option for reducing polyphenol and tannin levels, which are strongly associated with adverse effects that limit the use of protein isolates as food-grade ingredients, affecting their nutritional and sensory properties. However, further optimisation could be beneficial to reduce phytic acid content in DES-based protein isolates. Techniques such as combining DES-based extraction with enzymatic treatments, including the use of phytases to break down phytic acid, could be a promising approach to enhance the nutritional quality of the isolates.

### 3.4 Amino acid profile

The amino acid profile was determined to assess the protein quality and nutritional value of the protein isolates (Table 2). All amino acids were detected in the examined isolates, indicating that they have a beneficial nutritional value for human consumption and can be compared to some animal proteins, such as egg and milk (Chmielewska et al., 2021; Fleddermann et al., 2013).

**Table 2:** Amino acid profile of rapeseed protein isolates.

Amino acid (g/100 g sample)^a^	ALK12	DES1	DES2
Aspartic acid	7.11 ± 0.15^b^	7.76 ± 0.11^a^	5.45 ± 0.12^c^
Arginine	8.57 ± 0.11^a^	5.15 ± 0.13^b^	3.06 ± 0.12^c^
Serine	3.96 ± 0.08^b^	4.29 ± 0.05^a^	2.88 ± 0.09^c^
Tyrosine	2.06 ± 0.04^a^	1.85 ± 0.06^b^	1.66 ± 0.06^c^
Glutamine	13.9 ± 0.17^b^	18.1 ± 0.17^a^	8.43 ± 0.13^c^
Proline	2.99 ± 0.11^b^	3.22 ± 0.08^a^	0.35 ± 0.02^c^
Glycine	4.43 ± 0.10^b^	5.78 ± 0.10^a^	2.99 ± 0.08^c^
Alanine	3.66 ± 0.10^b^	4.08 ± 0.10^a^	3.05 ± 0.09^c^
Cystine	1.70 ± 0.17^b^	3.60 ± 0.06^a^	0.30 ± 0.04^c^
Valine	4.29 ± 0.10^b^	6.05 ± 0.13^a^	3.19 ± 0.07^c^
Methionine	1.29 ± 0.06^b^	2.47 ± 0.04^a^	0.87 ± 0.05^c^
Isoleucine	2.88 ± 0.09^b^	3.34 ± 0.07^a^	2.18 ± 0.07^c^
Leucine	5.25 ± 0.11^b^	6.39 ± 0.10^a^	3.90 ± 0.10^c^
Threonine	3.58 ± 0.08^a^	3.48 ± 0.03^a^	2.70 ± 0.07^b^
Phenylalanine	3.35 ± 0.07^b^	3.94 ± 0.10^a^	2.56 ± 0.07^c^
Histidine	1.83 ± 0.04^a^	1.73 ± 0.07^a^	1.15 ± 0.05^b^
Lysine	5.09 ± 0.13^a^	4.38 ± 0.10^b^	3.70 ± 0.10^c^

NEAA	48.3	53.8	28.2
EAA	27.6	31.8	20.3
n ratio	0.57	0.59	0.72

**TAA**	**75.9**	**85.6**	**48.5**

Different letters in each raw indicate significant (*p* > 0.05) difference between the isolates.

n.d. – not detected.

ALK12 – isolate obtained after alkaline extraction at pH 12.

DES1 – isolate obtained by DES extraction (choline chloride:urea = 1:2, 10 % H_2_O, pH 7.60).

DES2 – isolate obtained by DES extraction (betaine:citric acid = 1:1, 20 % H_2_O, pH 2.45).

NEAA – non essential amino acids; EAA – essential amino acids; TAA – total amino acids.

a On a dry matter basis.

The highest amino acid content was found in the DES1 isolate (85.6 g/100 g – Table 2), which correlates with its protein content (Table 1). The most abundant amino acids in all isolates were glutamine, arginine, and leucine, while methionine and histidine were present in the lowest amounts. These results align with the findings published by Aider and Barbana (2011). Sulphur-containing amino acids (methionine and cystine) exceeded 3 % in the DES1 and DES2 isolates, meeting the human nutritional requirements set by WHO/FAO/UNU. Their levels were also higher than those found in most vegetable proteins, including legumes (Alashi et al., 2013). These amino acids and their metabolites, such as glutathione and homocysteine, play a crucial role in antioxidant defense and the regulation of redox balance, making rapeseed isolates important for the human diet (Fleddermann et al., 2013). A study by Mnzava and Olsson (1990) reported lysine as the limiting amino acid, which contradicts the findings of this study. This discrepancy may be attributed to differences in rapeseed variety and processing methods, particularly since the defatting processes in the mentioned study involved high temperatures during oil extraction, which could lead to lysine degradation via the Maillard reaction (Newkirk et al., 2003).

Among the essential amino acids (EAA), leucine and lysine were the most abundant, with the highest total EAA content observed in DES1. The lowest essential-to-non-essential amino acid (EAA/NEAA) ratio was found in ALK12 (0.57), while the highest was observed in DES2 (0.72). In isolates obtained through DES-based extraction, the EAA/NEAA ratio was generally 0.6 or higher, aligning with FAO/WHO dietary recommendations based on age and physiological needs (World Health Organization, 2007). The amino acid composition of all investigated isolates provides valuable insights, highlighting rapeseed protein isolates as high-quality protein sources. In terms of amino acid composition, the protein isolates obtained through DES-based extraction are comparable to, or even superior to, those obtained through alkaline extraction (particularly in the case of DES1). Its well-balanced amino acid profile makes it especially suitable for vegan diets, offering an excellent plant-based alternative to animal proteins.

### 3.5 Isolate colour parameters

**Fig. 1:**
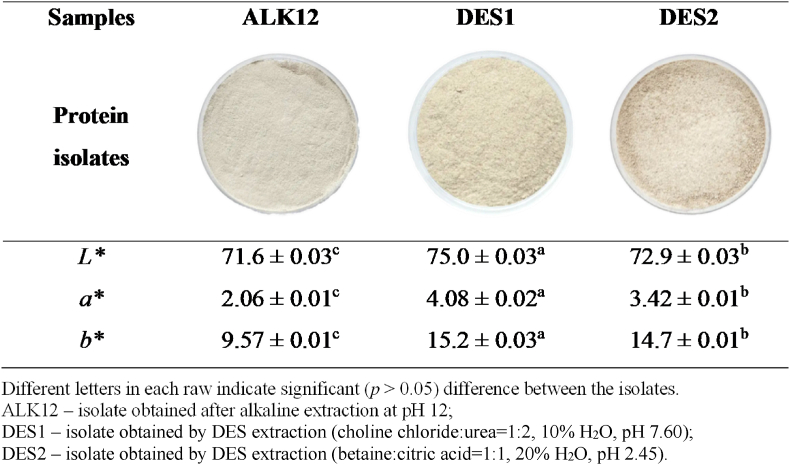
Colour parameters of rapeseed protein isolates.

Colour is an important characteristic of rapeseed protein isolates, as it influences their acceptability in food products, reflecting not only sensory properties but also nutritional quality and the degree of purification *(*Albe-Slabi et al., 2022*).* The colour parameters *(L∗, a∗,* and *b∗*) of the isolates, along with a visual representation, are presented in Fig. 1 to highlight differences in their shades.

All isolates exhibited a similar, desirable off-white colour, which can primarily be attributed to freeze-drying applied in their production. This method was selected because it enables the production of lighter isolates compared to vacuum drying (Hu et al., 2009; Shen et al., 2021).

Despite the isolates displaying a bright and uniform colour, the ALK12 isolate showed traces of a greenish hue, which was also confirmed by its lowest *a∗* value (Fig. 1)*.* The *L∗* value for all isolates exceeded 70, aligning with the findings of Karimi et al. (2024) for isolates obtained via NDES extraction. However, in this study, the isolate obtained through alkaline extraction at pH 12 exhibited a significantly (*p* ≤ 0.05) higher *L∗* value compared to the ALK12 isolate from the aforementioned study, which can be attributed to the removal of phenolic compounds through the applied pretreatment.

Although purification treatments can significantly lighten protein isolates, no process can completely eliminate residual colouration. Xu and Diosady (2002) reported that their isolate, even after combined treatment, retained a light brown colour with an *L* value of 69.3. The intensified colour of isolates obtained through alkaline extraction is primarily due to the presence of phenolic compounds and their oxidation products, which can form coloured complexes with rapeseed proteins via both covalent and non-covalent interactions, even at very low amounts (Zhang et al., 2024).

The *a∗* and *b∗* values obtained in this study were higher compared to the results of Karimi et al. (2024), which can be explained by the influence of ultrasonic treatment applied during pretreatment. Previous research has shown that ultrasound can induce changes in the colour of protein isolates by increasing the exposure of internal hydrophobic groups, leading to elevated *a∗* and *b∗* values (Zhou et al., 2015).

### 3.6 Protein molecular and structural characterisation

#### 3.6.1 Fourier-transform infrared spectrum (FT-IR)

**Fig. 2:**
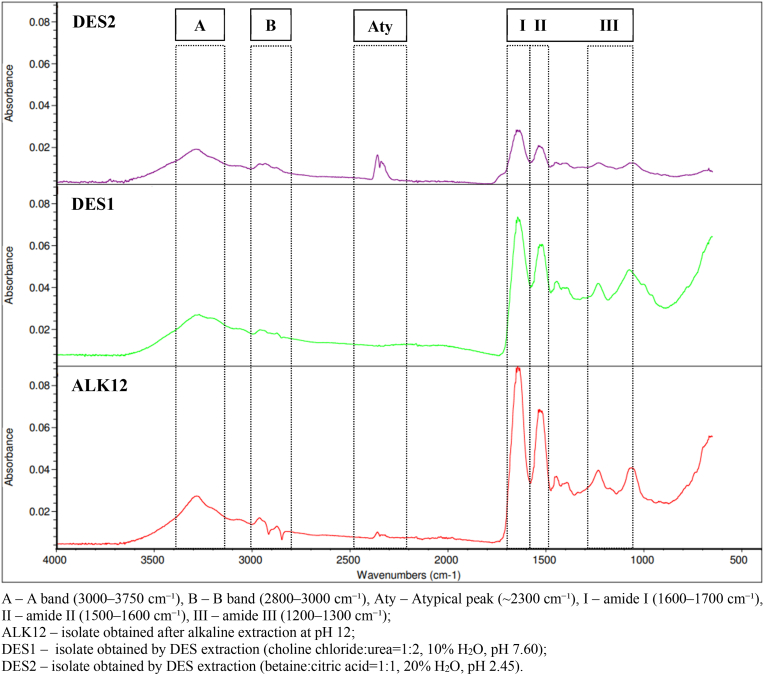
Fourier-transform infrared spectrum (FTIR) of rapeseed protein isolates.

FT-IR spectroscopy is an effective technique for assessing the secondary structure of proteins (Miller et al., 2013). Spectra in the range of 400–4000 cm^−1^ were analysed to identify the functional groups in the DES2, DES1, and ALK12 protein isolates (Fig. 2). The characteristic infrared absorption peaks observed include the amide A band (3000–3750 cm^−1^), amide B band (2800–3000 cm^−1^), amide I (1600–1700 cm^−1^), amide II (1500–1600 cm^−1^), and amide III (1200–1300 cm^−1^) bands. In the amide I region (1632 cm^−1^), corresponding to the C=O stretching of the amide group, a difference in absorbance was observed, with the lowest absorbance found for DES2 (Das Purkayastha et al., 2014; Glassford et al., 2013; Jiang et al., 2019). This suggests that the protein may have undergone denaturation or conformational changes, disrupting the peptide bonds responsible for the amide I peak (Das Purkayastha et al., 2014; Fu et al., 2024). Such changes can lead to a decrease in the intensity of the amide I band due to less defined or weakened C=O stretching vibrations. Additionally, denaturation may reduce the content of α-helix or β-sheet structures. A similar trend was observed with the amide II (1516 cm^−1^) and amide III (1218 cm^−1^) peaks, where lower absorbance values suggest changes in the secondary protein structure in the case of DES2. Both DES2 and ALK12 exhibited an atypical peak around 2300 cm^−1^, which could indicate the formation of new compounds. This may be attributed to the extreme pH conditions used in both extraction systems. In highly acidic or basic conditions, dissolved gases or newly formed ionic species (e.g., carbonate/bicarbonate in alkaline media) can produce IR absorptions in this region (Zhivulin et al., 2020). This signal is less common in neutral or near-neutral media (e.g., DES1), supporting the idea that extreme pH fosters such by-products or transient species (Karimi et al., 2024). The DES2 isolate exhibited more prominent conformational changes, which can induce protein unfolding by disrupting internal electrostatic bonds, possibly due to the acidic nature of the extraction medium and stronger ionic interactions. These observations suggest that extreme pH values can influence protein structure, even when DES is applied. The DES1 isolate, obtained using the DES system with a pH of 7.60, displayed a cleaner spectrum with characteristic protein peaks, aligning with its purity of 95.8 % (Table 1). This observation is consistent with the results obtained by Karimi et al. (2024), which show that pH-induced changes can significantly affect protein secondary structures.

#### 3.6.2 Lab-on-a-chip electrophoresis

Lab-on-a-chip electrophoresis was used to analyse the protein profile of the obtained protein isolates. Rapeseed proteins primarily consist of two abundant proteins, cruciferin and napin, typically found in concentrations of 55–85 % and 15–45 %, respectively (Chmielewska et al., 2021; Gerzhova et al., 2016). Cruciferin is a 300–350 kDa hexamer, composed of two trimers, with each monomer consisting of α (∼30 kDa) and β (∼20 kDa) polypeptides, linked by disulphide bonds (Karimi et al., 2024). The hexamer is stabilised by non-covalent interactions. In contrast, napin (12–16 kDa) is a monomer, composed of short (∼4 kDa) and long (∼9 kDa) polypeptides, also connected by disulphide bonds (Wanasundara et al., 2017).

**Fig. 3:**
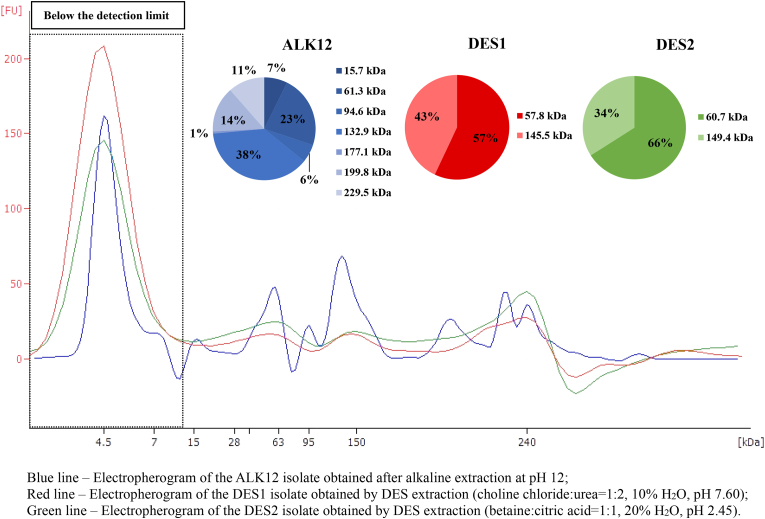
Lab-on-a-chip electrophoresis of rapeseed protein isolates.

As shown in Fig. 3, the DES1 and DES2 isolates exhibited similar protein profiles, which differed from those of the ALK12 isolate. Proteins extracted under alkaline conditions were separated into seven major bands, with estimated molecular weights of 15, 61, 94, 132, 177, 199, and 229 kDa. The most abundant fraction was 132 kDa, accounting for 38 % of the total protein content, with a concentration of 20.46 ng/μL. The second most prominent peak, at 61 kDa, accounted for 23 % of the total protein content, with a concentration of 13.73 ng/μL. This fraction likely corresponds to a cruciferin (12S globulin) subunit, which is in agreement with the findings of Chmielewska et al. (2021). Higher molecular weight fractions, including 177 kDa (4.8 ng/μL), 199 kDa (11.06 ng/μL), and 229 kDa (10.06 ng/μL), collectively accounted for 26 % of the total protein content. These bands likely correspond to cruciferin oligomers in various assembly states. Such oligomerisation may occur under extreme alkaline conditions (pH > 10), where structural interactions are disrupted, leading to partial unfolding into trimers or monomers. The exposure of hydrophobic regions further promotes protein aggregation (Karimi et al., 2024). The presence of a 15 kDa fraction suggests the possible occurrence of napin (2S albumin) or oleosin (Ahlström et al., 2022). Oleosin, commonly found in plant proteins, typically has a molecular weight ranging from 15 to 24 kDa (Rayner, 2015). The 94 kDa fraction may represent an intermediate protein complex or a cruciferin dimer.

The DES1 isolate exhibited two major bands at 57 kDa, accounting for 57 % of the total protein content (15.25 ng/μL), and 145 kDa, representing 43 % (13.97 ng/μL). The protein profile of DES2 was similar to DES1, with 66 % of the total protein content at 60 kDa and 34 % at 149 kDa. The 57 and 60 kDa fractions likely correspond to cruciferin subunits (12S globulin), while the 145 and 149 kDa fractions correspond to cruciferin oligomers. Notably, all isolates displayed high peaks at 4.5 kDa, which may be associated with the napin subunit, although this is below the detection limit of the kit used (Ahlström et al., 2022).

The broader distribution of higher molecular weight (MW) proteins observed in the DES1 and DES2 isolates, particularly the dominant bands around 145–149 kDa, suggests that these systems may preserve higher-order protein structures, such as cruciferin oligomers, and enable the extraction of structurally intact protein structures. DES1 likely facilitates protein solubilisation through hydrogen bonding and chaotropic interactions, promoting the maintenance of native-like quaternary structures. In contrast, DES2 may enable selective extraction of protein assemblies through electrostatic interactions and mild acid-assisted dissociation of protein-matrix complexes. Compared to alkaline extraction, which yielded a more fragmented profile characterised by several intermediate MW bands, the DES systems demonstrated a narrower and higher MW distribution, indicative of reduced denaturation and aggregation.

Although the extraction of higher MW proteins was not the explicit objective, the ability of DESs to preserve oligomeric structures such as cruciferin trimers or higher-order assemblies is noteworthy, particularly in cases where specific techno-functional properties of protein isolates, which are dependent on MW and oligomeric state, are desired (Zhang et al., 2020).

### 3.7 Protein functional characterisation

#### 3.7.1 Protein solubility

**Fig. 4:**
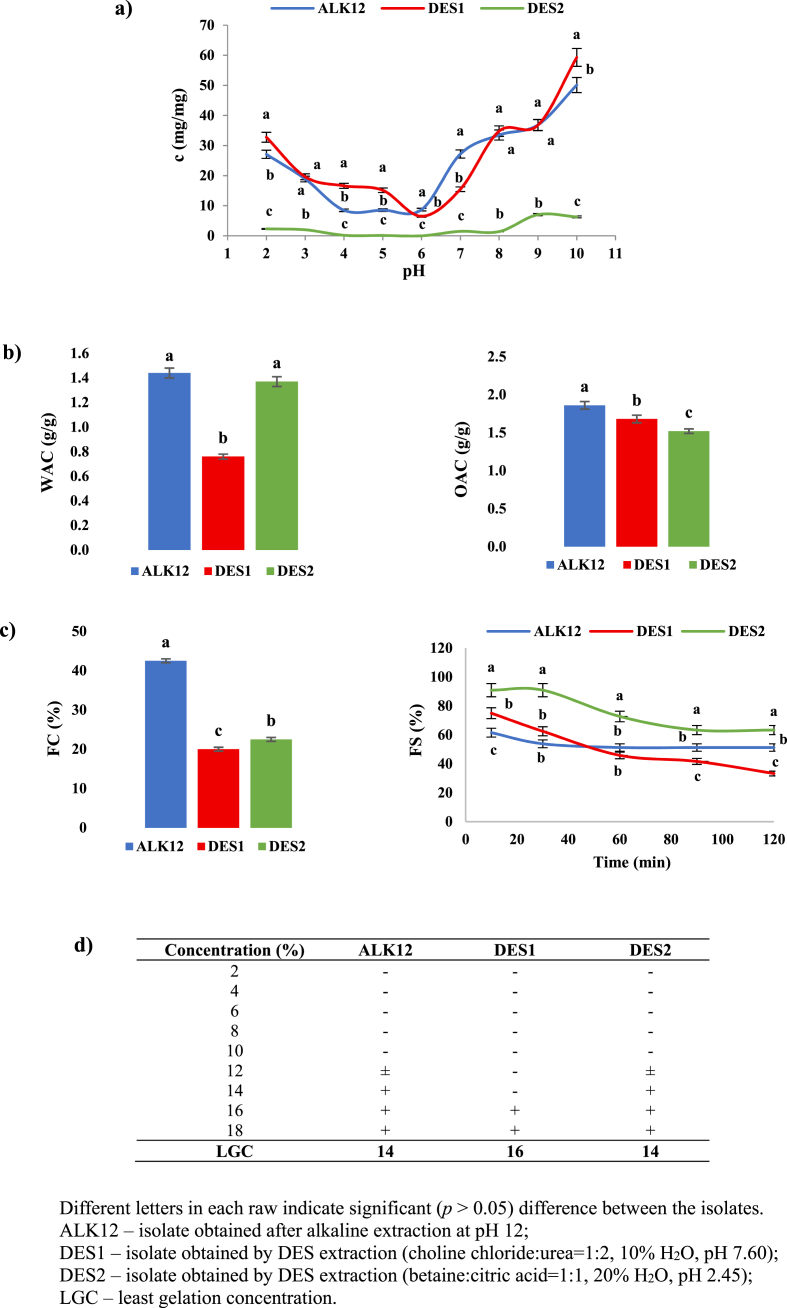
Techno-functional properties of rapeseed protein isolates: a) protein solubility, b) water absorption capacity (WAC) and oil absorption capacity (OAC), c) foaming capacity (FC) and foam stability (FS), and d) least gelation concentration (LGC).

Rapeseed protein isolates exhibit varying solubility profiles in aqueous media depending on their composition and extraction method (Fetzer et al., 2020). Fig. 4a illustrates the solubility profiles as a function of pH, ranging from 2 to 10, for protein isolates obtained through alkaline extraction (ALK12) and two different DES systems (DES1 and DES2). DES1 and ALK12 isolates exhibited significantly better solubility compared to DES2 across the entire tested pH range. These results can be attributed to the higher purity of DES1 and ALK12 isolates, which contain lower levels of non-protein components such as phytic acid, fibres, and other insoluble materials. These components, present in higher amounts in DES2 (29.7 %), can interfere with proteins and reduce their solubility. Moreover, higher solubility is attributed to partial denaturation in the alkaline medium, which disrupts the compact structure of the protein network and exposes more polar residues on the surface. This exposure enhances protein solubility by revealing polar groups while minimising the excessive exposure of nonpolar amino acids, which could otherwise trigger aggregation.

The high solubility observed under extremely acidic conditions (pH 2 and 3), followed by a decline in solubility as the pH increased to pH 4 and pH 6 for ALK12 and DES1, respectively, reflects a trend similar to that reported by Karimi et al. (2024). This pattern can be attributed to the closeness of the protein's isoelectric point to the acidic pH range. At pH values closer to the isoelectric point, proteins tend to have minimal net charge, leading to reduced solubility and an increased tendency to aggregate or precipitate. As the pH continued to rise, an increase in solubility was observed across all isolates, particularly beyond pH 6. This behavior suggests that the proteins regain a net charge in the alkaline range, which prevents aggregation and enhances their solubility. This phenomenon aligns with the expected properties of proteins, where solubility is typically higher in neutral to slightly alkaline conditions.

#### 3.7.2 Water and oil absorption capacity

Water absorption capacity (WAC) and oil absorption capacity (OAC) reflect the amphiphilic nature of proteins (Sibt-e-Abbas et al., 2020). WAC determines the ability of proteins to bind water, directly influencing the texture, stability, and rheological properties of food products. On the other hand, OAC is crucial for protein-lipid interactions, not only enhancing sensory attributes such as mouthfeel but also contributing to flavour retention, making it an important functional parameter in food products (Awuchi et al., 2019).

The results of this study showed that the highest WAC was exhibited by the isolate obtained via alkaline extraction (ALK12), with a value of 1.44 ± 0.04 g/g (Fig. 4b). A slightly lower WAC was recorded for the DES2 isolate (1.37 ± 0.04 g/g), while the lowest WAC was observed for the DES1 isolate, with a value of 0.76 ± 0.02 g/g (Fig. 4b). A similar trend was noted for OAC, where the highest value was observed for ALK12 (1.86 ± 0.05 g/g). A slightly lower, yet still significant, OAC was recorded for DES1 (1.68 ± 0.05 g/g) and DES2 (1.52 ± 0.03 g/g) (Fig. 4b).

Although a growing number of studies have explored the use of DESs for the extraction of alternative proteins, only a limited number have focused on their techno-functional properties, such as WAC and OAC. Cao et al. (2023) determined the WAC and OAC of proteins obtained from sesame meal; however, their results are not directly comparable to ours, as they used different DES systems. In the study by Arulrajah et al. (2025), protein extraction from kenaf seeds was performed using alkali, choline chloride-urea, and choline chloride-glycerol systems, with alkali-extracted proteins exhibiting significantly higher WAC and OAC, similar to our findings.

The differences in WAC and OAC among the rapeseed protein isolates can be attributed to two main factors. The first is the composition of the isolates – the presence of proteins, carbohydrates, and other components can influence their ability to bind water and oil. In this study, it can be assumed that the pretreatment with ethanol for polyphenol removal may have also altered the structure of the isolates, potentially forming a more rigid matrix that hindered hydration and reduced their ability to bind water and oil (Jia et al., 2021).

Additionally, considering the primary goal of this study, which was to investigate two DES systems with distinct pH values, it is important to note that varying water contents likely influenced the properties of these systems, particularly in terms of WAC. The addition of water to DESs was primarily intended to modulate pH, but it also altered the intermolecular hydrogen bonding interactions between hydrogen bond donors and acceptors. This likely increased the polarity of DESs (Dai et al., 2015; Hanafi et al., 2024), enhancing the extraction of more polar proteins and, consequently, increasing the WAC of the obtained protein isolates. This is supported by the observation that DES2, with 20 % water added to the DES-based system, exhibited a WAC approximately twice as high as that of DES1, which contained only 10 % water. These findings suggest that the addition of water to DES systems can significantly influence the WAC of the extracted proteins.

In general, the observed differences in the techno-functional properties of proteins resulting from various extraction methods highlight the importance of process optimisation to achieve tailored products with specific characteristics. Future studies should focus on optimisation strategies that consider multiple output factors, where the selection of optimal procedures is guided by a careful assessment of their significance, such as balancing phenolic compounds removal against potential reduction in WAC. This approach will contribute to the development of more efficient and customised extraction techniques for specific applications.

#### 3.7.3 Foaming capacity and stability

Fig. 4c illustrates the foaming capacity (FC) and foam stability (FS) of protein isolates over 2 h at pH 7. The highest foaming capacity was recorded for the isolate obtained through alkaline extraction (42.5 ± 0.50 %), while significantly (*p* ≤ 0.05) lower values were observed for the DES1 and DES2 isolates, amounting to 20.0 ± 0.50 % and 22.5 ± 0.50 %, respectively. Karimi et al. (2024) reported different findings, where the highest FC values were recorded for NDES isolates compared to those obtained through alkaline extraction. This difference could be attributed to the ultrasound treatment used in the pretreatment for polyphenol removal in our study, as Li et al. (2021) found that microwave or ultrasound treatment could disrupt the fundamental cellular and tissue structures of rapeseed, leading to the formation of proteins with lower molecular weights, which are responsible for the lower FC values.

Regarding foam stability, the ALK12 isolate exhibited the smallest change in foam volume over time, maintaining a stable value after an initial slight decrease. The DES1 isolate showed the lowest stability, with a pronounced decline in FS values throughout the testing period. In contrast, the DES2 isolate initially demonstrated high foam stability, with a gradual decrease over time, yet it still retained better foam stability compared to DES1 (Fig. 4c). These results suggest that extraction methods significantly influence not only the initial foaming capacity but also the long-term foam stability.

#### 3.7.4 Gelling ability

Rapeseed proteins have so far exhibited limited gelation ability, with stronger gels consistently reported at higher temperatures and pH values, likely due to increased protein unfolding and subsequent molecular interactions (Fetzer et al., 2020). Khattab and Arntfield (2009) determined that rapeseed requires a higher least gelation concentration (LGC) compared to sesame and flaxseed, regardless of the applied treatment.

Our results showed that the minimum concentration required for gelation was 14 % for ALK12 and DES2, while for DES1, it was 16 % (Fig. 4d). However, gel formation was observed at 12 % for ALK12 and DES2, though its structure changed over time, with a stable gel detected at 14 %. In contrast, for DES1, a stable and firm gel was formed only at 16 % isolate concentration. Similar findings were reported by Sibt-e-Abbas et al. (2020), who observed that rapeseed protein isolates formed a stable gel at 14 % concentration. The differences in gelation ability among the investigated protein isolates can be attributed to variations in their protein content (Sibt-e-Abbas et al., 2020), with DES1 exhibiting a significantly higher protein content compared to ALK12 and DES2.

### 3.8 *In vitro* digestibility

Rapeseed protein isolates represent a significant source of protein, but their nutritional value depends on their degree of digestibility. Protein digestion is crucial for assessing bioavailability, with *in vitro* enzymatic methods commonly used to simulate the human digestive tract under controlled pH conditions and a constant temperature, utilising specific enzymes. The goal of *in vitro* digestion is to evaluate protein digestibility and potential bioactivity of peptides released after consumption. Protein digestibility is influenced by their structural characteristics, solubility, interaction with other components, and the presence of antinutritional factors (Joehnke et al., 2018; Wanasundara et al., 2016). Namely, phytates and phenolic acids can inhibit proteolytic enzyme activity and form complexes with proteins, reducing their availability for enzymatic hydrolysis (Bailey et al., 2023).

In this study, the digestibility of protein isolates obtained through alkaline and DES-based extraction was analysed. Digestibility was assessed using the standardised INFOGEST protocol (Minekus et al., 2014). Protein molecular distribution before and after digestion was assessed via lab-on-chip electrophoresis with results expressed as the relative abundance of detected protein fractions. The characterisation of the protein profile enables the monitoring of digestion efficiency and the degree of hydrolysis of large protein structures into smaller, more bioavailable peptides, offering insight into the nutritional quality of the extracted proteins.

**Fig. 5:**
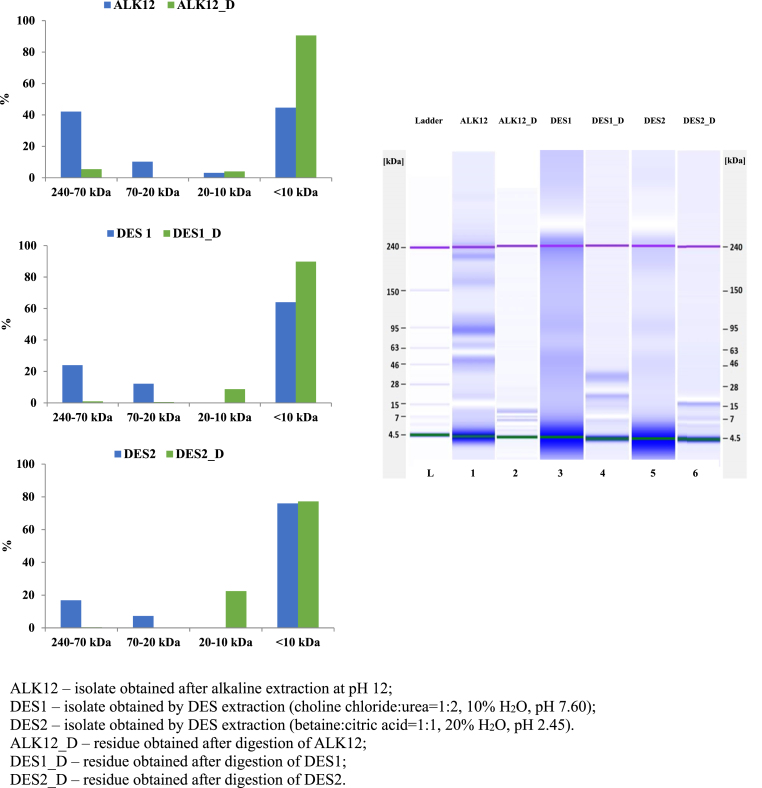
Molecular mass distribution in rapeseed protein isolates before and after digestion.

The graphical representation (Fig. 5) of protein molecular distribution before and after digestion was structured to segment protein fractions based on molecular weight ranges, ensuring the identification of key storage proteins and their degradation patterns. The 240–70 kDa range primarily captures the large fractions of cruciferin, whose subunit composition can vary depending on pH and ionic strength. The 70–20 kDa range includes cruciferin monomer (48–56 kDa), α-unit (30–40 kDa), and β-unit (∼20 kDa), which play an essential role in the functional properties of protein isolates (Ntone et al., 2022; Wanasundara et al., 2016). The 20–10 kDa range primarily represents larger napin fractions which exist as a heterodimer (∼14–17 kDa) (He et al., 2021). Fractions <10 kDa encompass smaller napin fragments, low-molecular-weight peptides, and free amino acids, which result from extensive enzymatic hydrolysis. These smaller peptides are crucial for evaluating protein digestibility and bioavailability and may contribute to bioactive properties (Mundi and Aluko, 2012, Tan et al., 2011).

Prior to digestion, the ALK12 isolate exhibited a nearly balanced distribution of cruciferin and short (∼4 kDa) napin fractions, with proteins corresponding to the cruciferin fraction accounting for 52.34 % of the total peak area. After digestion, the proportion of non-digested cruciferin fractions decreased to 5.43 %, highlighting its high susceptibility to enzymatic hydrolysis in the ALK12 isolate. The fraction of peptides below 10 kDa significantly increased, indicating extensive proteolytic degradation. By the end of the digestion process, peptides below 10 kDa accounted for 90.57 % of the total peak area, demonstrating the breakdown of high-molecular-weight proteins into smaller peptides and free amino acids. This extensive hydrolysis suggests that the ALK12 isolate exhibited a high degree of digestibility and potential bioactive functionality.

In comparison to ALK12, the dominant fractions in the DES1 and DES2 isolates were short (∼4 kDa) napin fractions, accounting for 63.96 % and 75.94 % of the total protein peak area, respectively. This aligns with the fact that napin is soluble within the pH ranges of both DES systems, considering it is a strongly basic protein with an isoelectric point around pH 11 (Akbari and Wu, 2015). An increased proportion of napin, which breaks down into smaller peptides, can enhance absorption and potentially offer health benefits, particularly through bioactive peptides with antihypertensive and antioxidant properties (Wanasundara et al., 2016).

In terms of digestibility, DES1 demonstrated significantly better results, comparable to ALK12. A marked increase in the proportion of fractions smaller than 10 kDa was observed, reaching 89.77 % after digestion. In contrast, DES2 retained a much higher proportion of fractions between 10 and 20 kDa (22.43 % compared to 8.71 % in DES1), suggesting that DES1 facilitated more efficient proteolytic degradation.

This superior digestibility in DES1 can be attributed not only to its higher purity but also to its enhanced solubility across a broad pH range (Fig. 4). During *in vitro* digestion, proteins are first exposed to acidic gastric conditions and then to alkaline conditions during the intestinal phase. Given that DES1 maintained good solubility in both environments, enzymatic hydrolysis was likely more efficient, allowing for greater accessibility of enzymes to the protein substrate and, consequently, a significantly higher increase in fractions below 10 kDa.

## 4 Conclusions

This study provides a detailed comparative analysis of protein isolates derived from defatted rapeseed cake using conventional alkaline extraction (ALK12) and two deep eutectic solvent (DES)-based systems (choline chloride:urea and betaine: citric acid) as green extraction alternatives. The results demonstrate that the ALK12 isolate achieved the highest protein extraction efficiency (36.9 %), while the DES1 achieved the highest protein purity (95.8 %). Despite its excellent techno-functional properties, the highest phenolic content in the ALK12 isolate may negatively impact the nutritional quality and sensory attributes.

DES1 extraction, although characterised by a lower yield (23.2 %), resulted in the highest protein purity (95.8 %) and a significantly reduced content of phenolic compounds compared to ALK12. Importantly, the DES1 isolate exhibited the highest content and favourable amino acid composition, meeting FAO/WHO recommendations for essential amino acid intake, and demonstrated excellent solubility across a broad pH range, which translated into enhanced enzymatic digestibility and a high proportion of low-molecular-weight peptides following *in vitro* gastrointestinal simulation. These characteristics make DES1 isolate particularly suitable for of high-protein functional food formulations.

DES2 offered a moderate compromise between extraction yield and nutritional quality but was limited by lower solubility.

Overall, the findings underscore the importance of tailoring the extraction method to maximise yield and optimise the compositional, structural, and functional attributes of the resulting protein isolates. DES-based systems, particularly those operating at near-neutral pH, such as DES1 system, represent a promising and sustainable approach for plant protein extraction. Further comprehensive, multi-parameter optimisation could refine and enhance the nutritional quality and techno-functional properties of DES-based proteins. Future research should also address scale-up feasibility and evaluate the performance of the resulting protein isolates in real food applications.

## CRediT authorship contribution statement

**Branislava Đermanović:** Investigation, Formal analysis, Writing – original draft. **Aleksandar Marić:** Conceptualization, Methodology, Writing – original draft. **Marijana Sakač:** Conceptualization, Writing – original draft, Writing – review & editing. **Damjana Tomić:** Data curation, Formal analysis, Validation. **Danka Dragojlović:** Investigation, Data curation, Writing – original draft. **Ljiljana Popović:** Conceptualization, Validation, Visualization. **Bojana Šarić:** Conceptualization, Validation, Writing – review & editing. **Pavle Jovanov:** Conceptualization, Supervision, Project administration.

## Declaration of competing interest

The authors declare that they have no known competing financial interests or personal relationships that could have appeared to influence the work reported in this paper.

## Data Availability

Data will be made available on request.
